# Resin-Immobilized Palladium Nanoparticle Catalysts for Organic Reactions in Aqueous Media: Morphological Aspects

**DOI:** 10.3390/molecules201018661

**Published:** 2015-10-14

**Authors:** Piero Mastrorilli, Maria M. Dell’Anna, Antonino Rizzuti, Matilda Mali, Mauro Zapparoli, Cristina Leonelli

**Affiliations:** 1 Dipartimento di Ingegneria Civile, Ambientale, del Territorio, Edile e di Chimica (DICATECh), Politecnico di Bari, via Orabona 4, Bari 70125, Italy; E-Mails: mariamichela.dellanna@poliba.it (M.M.D.A.); antonio.rizzuti@poliba.it (A.R.); matilda.mali@poliba.it (M.M.); 2 Centro Interdipartimentale Grandi Strumenti (C.I.G.S.), Università di Modena e Reggio Emilia, via Campi 213\A, Modena 41125, Italy; E-Mail: mauro.zapparoli@unimore.it; 3 Dipartimento di Ingegneria “Enzo Ferrari”, Università di Modena e Reggio Emilia, via Pietro Vivarelli 10, Modena 41125, Italy; E-Mail: cristina.leonelli@unimore.it

**Keywords:** polymer supported Pd nanoparticles, morphological analysis, TEM, micro-IR spectroscopy, recyclable catalyst, quinoline transfer hydrogenation, water solvent, nitrobenzene reduction, quinoline hydrogenation, Suzuki-Miyaura coupling

## Abstract

An insight into the nano- and micro-structural morphology of a polymer supported Pd catalyst employed in different catalytic reactions under green conditions is reported. The pre-catalyst was obtained by copolymerization of the metal-containing monomer Pd(AAEMA)_2_ [AAEMA^−^ = deprotonated form of 2-(acetoacetoxy) ethyl methacrylate] with ethyl methacrylate as co-monomer, and ethylene glycol dimethacrylate as cross-linker. This material was used in water for the Suzuki-Miyaura cross-coupling of aryl bromides, and for the reduction of nitroarenes and quinolines using NaBH_4_ or H_2_, as reductants. TEM analyses showed that in all cases the pristine Pd(II) species were reduced *in situ* to Pd(0), which formed metal nanoparticles (NPs, the real active species). The dependence of their average size (2–10 nm) and morphology on different parameters (temperature, reducing agent, presence of a phase transfer agent) is discussed. TEM and micro-IR analyses showed that the polymeric support retained its porosity and stability for several catalytic cycles in all reactions and Pd NPs did not aggregate after reuse. The metal nanoparticle distribution throughout the polymer matrix after several recycles provided precious information about the catalytic mechanism, which was truly heterogeneous in the hydrogenation reactions and of the so-called “release and catch” type in the Suzuki coupling.

## 1. Introduction

The value of nanoparticles (NPs) is one of the discoveries that has recently been developed to an extent that scientists could have never imagined a century ago, also in the field of catalysis [1,2,3,4]. The main problem arising with the use of metal nanoparticles as catalysts is their tendency to form agglomerates, which decreases their activity. To avoid aggregation, NPs are usually stabilized by inorganic or polymeric supports. Several methods are used nowadays to prepare metallic NPs entrapped in polymeric supports for use in gas sorption, separation, and heterogeneous catalysis [5]. Recently, it has been proven that high thermal and chemical stability can be attained when the polymer frame is made up of tunable building units with only covalent bonds [6,7,8]. Additionally, it has been reported that to avoid nanoparticle aggregation as in chemical vapor deposition [9,10,11,12], solid grinding [13,14,15,16,17], impregnation [18,19], and immobilization [20,21,22,23], the NP formation should occur concurrently with the reticulation of polymer matrices [24,25]. An alternative way to prepare highly dispersed polymer stabilized nanoparticles consists in the copolymerization of metal-containing monomers with suitable co-monomers [26], followed by reduction of the supported metal. In this field, for example Pomogailo *et al.* [27] carried out the thermal polymerization of Pd(H_2_C = CHCONH_2_)(NO_3_)_2_ in the presence of inorganic supports, such as SiO_2_, Al_2_O_3_, C, obtaining hybrid polymer inorganic composites. Pd(II) was then reduced under H_2_ to give supported Pd NPs. In this procedure, the preparation of the suitable Pd organometallic monomer to be linked with co-monomer units is critical, and a second reduction treatment was necessary. A reduced number of monomers could be easily bonded to Pd ion ligands, but when the ligands themselves could reticulate in a stable polymer, the preparation of the Pd NP catalyst was successful [28,29,30,31,32]. The convenience of NP formation concurrent with monomer reticulation has undoubtedly important implications as far as the process sustainability is concerned; the choice of cross-linking ligands is an additional step forward, but the lowest environmental impact in the catalytic process can be reached when water substitutes for the organic solvent as reaction medium. In fact, water has been recently viewed as an eco-friendly alternative to common organic solvents because it is non-toxic, non-flammable, has a high heat capacity, is cheap and renewable. In some cases, due to its high polarity, its acid-base properties and its hydrogen bonding ability, water may influence the reactivity and the selectivity of certain catalytic systems. However, sometimes the use of neat water depresses the catalytic efficiency, as it has been reported, for example, for Pd NPs stabilized by various polymers, such as poly(*N*-vinyl-2-pyrrolidone) [33,34,35], polystyrene-β-poly(sodium acrylate) [36] and poly(*N*,*N*-dihexylcarbodiimide) [37] in the Suzuki-Miyaura reaction. In many cases, the polymeric support does not swell in water, thus hampering the migration of the reactants to the active sites. Neverthless, a number of suitable polymeric matrices have been developed so far for C–C couplings [38], also under green conditions [39].

In other cases (e.g., in the selective reduction of quinolines), the formation of hydrogen bonds between the substrate and the water hydroxyl groups may inhibit the absorption of the reagent on the surface of the catalyst, thus lowering its activity [40]. For this reason, despite the advantages of using water as solvent, only a limited number of heterogeneous catalysts [41,42,43,44] have been employed for promoting the hydrogenation reactions of quinolines in water.

**Scheme 1:**
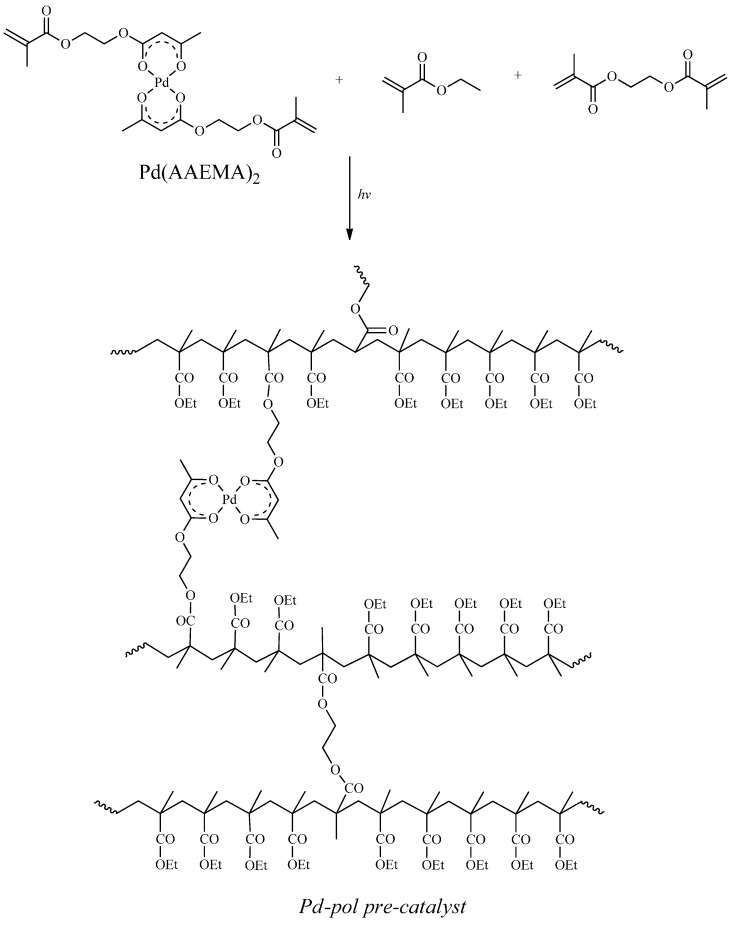
Synthesis of *Pd-pol* pre-catalyst.

The authors have already described a palladium based pre-catalyst for the Suzuki-Miyaura cross-coupling [45] as well as for the reduction of nitroarenes [46] and quinolines [47,48] in water as solvent. In order to obtain a material with a uniform distribution of the catalytically active sites, the pre-catalyst was not synthetized by classical immobilization of palladium centres onto a prefabricated support, but it was synthetised in a rather unconventional way, by co-polymerization of the metal-containing monomer Pd(AAEMA)_2_ [AAEMA^−^ = deprotonated form of 2-(acetoacetoxy)ethyl methacrylate] with a suitable co-monomer (ethyl methacrylate) and cross-linker (ethylene glycol dimethacrylate) (*Pd-pol* pre-catalyst, Scheme 1) [28,29]. *Pd-pol* was then reduced *in situ* under reaction conditions forming Pd NPs (the catalytically active species) immobilized and stabilized by the reticular and macro porous polymeric support. It is known that the properties of the polymeric support playing a crucial role in catalysis are the swelling capability, the porosity and a mild reticular structure [49]. Our *Pd-pol* swells in water and in many organic solvents, is macroporous and is characterised by a rather low degree of cross-linking, thus it was found active and recyclable for several palladium catalysed reactions [50,51,52,53,54,55] in conventional organic solvents as well as in water [56].

In this work, the catalyst recovered after several cycles of the Suzuki-Miyaura cross-coupling and of the hydrogenation of organic substances carried out in water, was studied in depth from the morphological point of view by high resolution transmission electron microscopy (HRTEM), electron diffraction and micro-IR spectroscopy.

The structural and chemical properties of *Pd-pol* before and after its use in catalysis, that is before and after Pd(II) reduction, were already addressed in our previous work [29] but a real-time monitoring of the evolution of the Pd NPs during the reduction process was missing, and the effect of different reducing agents (temperature, phenyl boronic acid, dihydrogen, NaBH_4_) was never explored. The *in situ* investigation of Pd NP formation is of great interest because it is well-known that size and shape of metal NPs depend on the kinetics of the nucleation and growth processes [57]. Hence, understanding the phenomena behind NP formation is fundamental in order to tune the NP properties, and therefore their catalytic performances [58].

In addition, our interest in investigating the re-used catalyst stemmed from the observation that the catalyst stability still represents the weak point of the heterogeneous catalysis that demands the reuse of the material even when reaction conditions are harsh.

## 2. Results and Discussion

### 2.1. Catalytic Efficiency and Re-Use

#### 2.1.1. Suzuki-Miyaura Cross-Coupling

*Pd-pol* was active, selective and recyclable in the Suzuki-Miyaura cross-coupling of aryl bromides and activated aryl chlorides in water at 100 °C in the presence of K_2_CO_3_ [45]. Table 1 reports the catalytic results obtained in the Suzuki coupling between 4-bromoacetophenone and phenyl boronic acid taken as a model reaction. The use of a transfer phase additive, such as tetrabutylammonium bromide (TBAB) [59] accelerated the reaction (Table 1, entry 2) for two reasons: it facilitated the dissolution of the organic reactants in the water phase and it permitted the formation of smaller size (more active) nanoparticles (see below).

**Table 1:** Suzuki reaction of 4-bromoacetophenone and phenylboronic acid in water under air over five cycles ^a^. 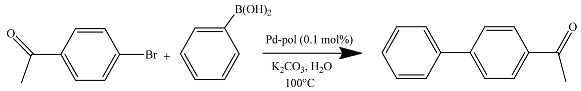

Entry	Time	Cycle (% Yield)				
		First	Second	Third	Fourth	Fifth
1	2 h	97	94	84	82	82
2 ^b^	15 min	98	93	85	84	80

^a^: 4-bromoacetophenone (1.0 mmol), phenylboronic acid (1.5 mmol), base (2.0 mmol), H_2_O (4.0 mL), T = 100 °C; ^b^: in the presence of TBAB (1.0 mmol).

**Scheme 2:**
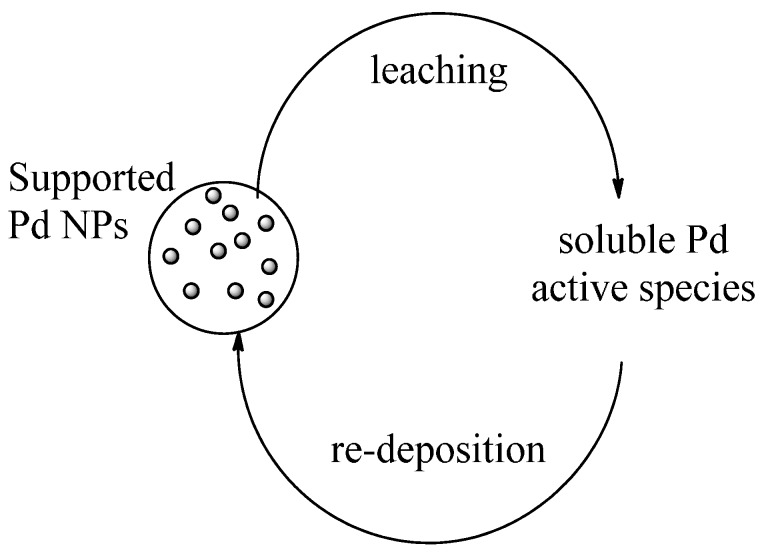
“Release and catch” mechanism for a Pd NPs based catalytic system.

Investigations on the activity of the mother liquor collected during and after catalytic runs (the mother liquor was catalytically active during the catalytic run, resulting inactive when collected after substrate consumption), ICP analyses carried out on the mother liquors and on the supported catalyst during and after the catalytic runs (Pd amount was negligible in the mother liquor recovered at end of reaction and was always the same in *Pd-pol* recovered after each catalytic cycle) (see [45]) revealed that *Pd-pol* could act as a “release and catch” catalytic system [60,61]. A “release and catch” catalytic system is prepared by noncovalent immobilization of the catalytic moiety onto a suitable support, but differently from the usual non-covalently supported catalyst, the catalytic moiety is released in solution during the course of the reaction and it is recaptured at the end of the reaction. Such a “catalyst-sponge like” system (Scheme 2) permits to combine the benefits of homogeneous (high catalytic activity) and heterogeneous catalysis (easy separation and recycling).

#### 2.1.2. Hydrogenation and Transfer Hydrogenation of Organic Compounds

Differently from the case of Suzuki-Miyaura coupling, *Pd-pol* behaves as a truly heterogeneous catalyst in nitroarene reduction in water (Table 2) [46], as well as in the selective hydrogenation of quinolines in aqueous medium under 10 bar H_2_ at 80 °C (Table 3) [47] or at 60 °C in the presence of NaBH_4_ [48] as hydrogen source (Table 4).

**Table 2:** Transfer hydrogenation of nitrobenzene over twelve cycles ^a^. 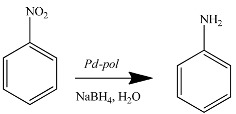

Cycle	1st	2nd	3rd	4th	5th	6th	7th	8th	9th	10th	11th	12th
**Yield (%)**	96	95	96	97	94	93	94	95	96	95	94	96

^a^: Substrate (1.0 mmol), H_2_O (5 mL), NaBH_4_ (10.0 mmol), *Pd-pol* (0.0175 mmol of Pd), room temperature, 2 h.

**Table 3:** Hydrogenation of 8-methylquinoline to 8-methyl-1,2,3,4-tetrahydroquinoline over nine cycles ^a^. 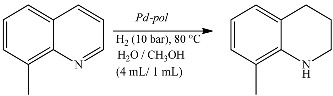

Cycle	First	Second	Third	Fourth	Fifth	Sixth	Seventh	Eighth	Ninth
**Yield (%)**	99	98	97	98	98	99	97	98	98

^a^: Substrate (1.0 mmol), H_2_O/CH_3_OH (4 mL/1 mL), *Pd-pol* (Pd: 0.5 mol %), 80 °C, 10 bar H_2_, 9 h.

**Table 4:** Transfer hydrogenation of quinoline to 1,2,3,4-tetrahydroquinoline over seven cycles ^a^. 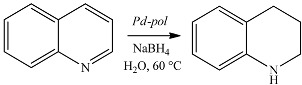

Cycle (% yield)							
Cycle	First	Second	Third	Fourth	Fifth	Sixth	Seventh
**Yield (%)**	94	94	95	92	91	93	94

^a^: Substrate (1.0 mmol), H_2_O (5 mL), NaBH_4_ (20.0 mmol), *Pd-pol* (Pd: 2.0 mol %), 60 °C, 9 h.

Interestingly, by carrying out the nitrobenzene hydrogenation using H_2_ (1 atm) as reducing agent instead of NaBH_4_, the reaction reached completion after 6 h in the first run and after 24 h in the second run, revealing that *Pd-pol* catalytic system was more active and recyclable using NaBH_4_.

In all reported reductions, the mother liquors hot filtered at 40% of substrate conversion were catalytically inactive, and the supported catalyst recovered at the end of the first run and at 40% conversion, respectively, contained the same amount of palladium as the pre-catalyst. The same palladium content was also found in the catalyst recovered at the end of the last cycle of the reduction reaction. These results rule out any possible contribution of homogeneous catalysis by leached palladium species and demonstrate that the polymer matrix was able to retain all the Pd NPs.

**Scheme 3:**
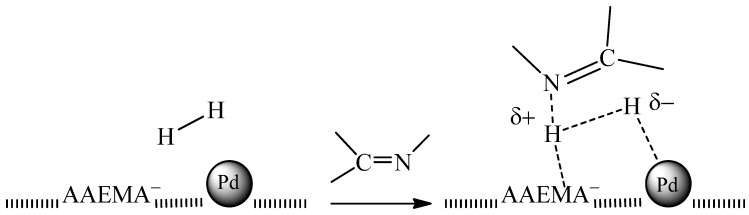
Heterolytic hydrogen splitting and ionic hydrogenation of polar substrates by Pd NPs on AAEMA^−^ supporting polymer matrix.

In addition, the polymer matrix could be involved in the mechanism of the reduction. In fact, the negative electrostatic charge on the surface of the support derived by the presence of AAEMA^−^ moieties (Scheme 1), could favor the heterolytic cleavage of H_2_ (used as the reductant or formed *in situ* by NaBH_4_) (Scheme 3), and the resulting H^+^/H^−^ pair could preferentially transfer to the C = N or N = O polar bonds [62]. The heterolytic splitting of H_2_ (assisted by the basic support) has been substantiated in some heterogeneous quinoline hydrogenation [63,64,65].

### 2.2. Micro-IR Analysis

Numerous investigations concerning incorporation techniques of metal nanoparticles into the polymer matrix have been published, demonstrating the stabilization of the metal nanoparticles encapsulated in the polymer matrix [58,66,67,68]. The approach used for the preparation of *Pd-pol* [26], starting from a polymerizable Pd(II) β-ketoesterate complex [29], is particularly interesting in catalysis applications due to the attainment of an intimate contact of the components of the composite material.

**Figure 1:**
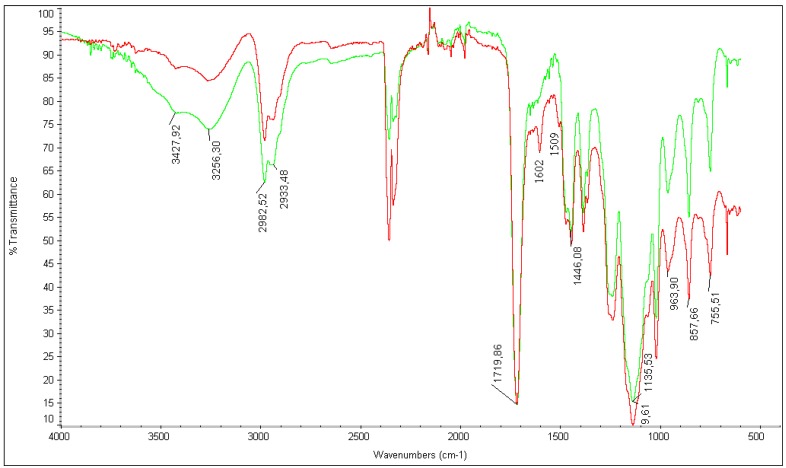
Micro-IR spectra of *Pd-pol* before (**red line**) and after (**green line**) 7 cycles of a NaBH_4_ reduction of quinoline.

Micro-IR spectroscopy analyses of the pristine *Pd-pol* and of the *Pd-pol* recovered after seven cycles of a NaBH_4_ reduction of quinoline (under the reaction conditions reported in Table 4) were performed, with the aim of gaining insight into the structural stability and the chemical modifications of the matrix after a catalytic process. IR spectra recorded before and after duty (Figure 1) are nearly superimposable, indicating that the polymer matrix backbone is stable. The disappearance of the weak β-ketoesterate combination band signals (1602 cm^−1^ and 1509 cm^−1^) [28] on passing from the pristine to the used catalyst is ascribable to the loss of metal coordination by the ligand, as a consequence of the reduction of the palladium oxidation state from +2 to zero that triggers the formation of NPs. These NPs may be stabilized by electro-static interactions with the oxygen atoms of the polymeric support [69].

### 2.3. TEM Observations

Transmission electron microscopy (TEM) has opened up new possibilities to investigate the nanoscale features of innovative catalysts with complex molecular structures via morphological and chemical characterization. The technique can be used in the *ex situ* approach when the catalyst is separated from the solution allowing a vast array of synthetic parameters to be varied without concern for the delicacies of the TEM equipment. In this respect, careful catalyst separation from the reaction media whereby the state of the nanoscopic features at the crystalline surface may be retained before transfer to the TEM can be performed. This is crucial to prevent secondary processes caused by changing growth conditions upon contact with air and extraction from the liquid media. TEM *ex situ* observations of nanoparticles in organic support must be accompanied by an accurate sample thinning operation to prevent contamination, deformation and particles release. When these requirements are accomplished, TEM gives a more direct approach to determining distribution, growth and dissolution rates of metal NPs onto the catalyst surface than the indirect reaction conversion rate or yield.

#### 2.3.1. Pristine *Pd-Pol*

The thermal co-polymerization of Pd(AAMA)_2_ resulted in the pre-catalyst (Scheme 1) characterized by an atomic Pd(II) polymeric network. However, the formation of primary Pd NPs could also occur, albeit in low extent, during the thermal decomposition of metal-containing precursors, in accordance with the simulated process proposed by Rozenberg *et al.* [30].

**Figure 2:**
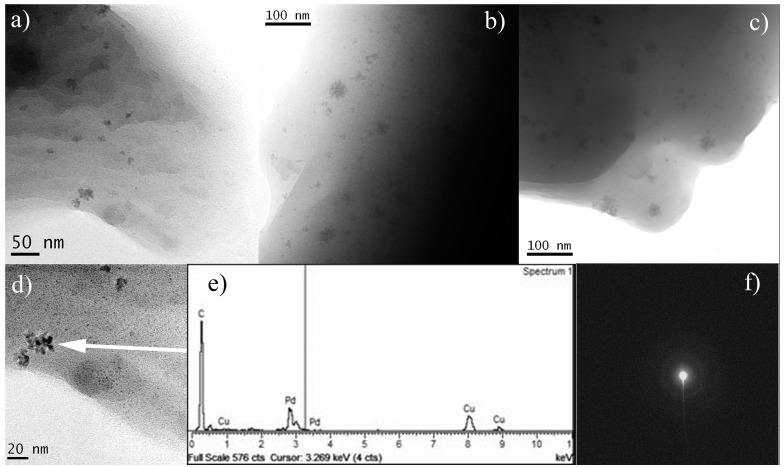
(**a**–**c**) TEM bright field images of three different batches of the pristine catalyst slice with PdNPs; (**d**) enlargement of a selected area of (**a**) accompanied by the corresponding (**e**) EDS spectrum and (**f**) SAED pattern.

TEM images of three different batches of *Pd-pol* pre-catalyst showing the morphological structure are reported in Figure 2a–c. At high magnification, all pristine catalysts showed almost the same microstructure consisting of a small number of round irregular polyhedral metallic Pd primary particles of *ca.* 9 nm, gathered in small aggregates with a maximum diameter of 18–25 nm and embedded in the polymeric matrix. This confirms the reproducibility of the pre-catalyst microstructure after preparation by a thermal polymerization process. The elemental identity of the nanoparticles was determined by means of Energy Dispersive Spectroscopy (EDS) and Selected Area Electron Diffraction (SAED) analyses. SAED image apparently consists of a ring pattern due to the diffraction of the thin metallic Pd-crystallites.

**Figure 3:**
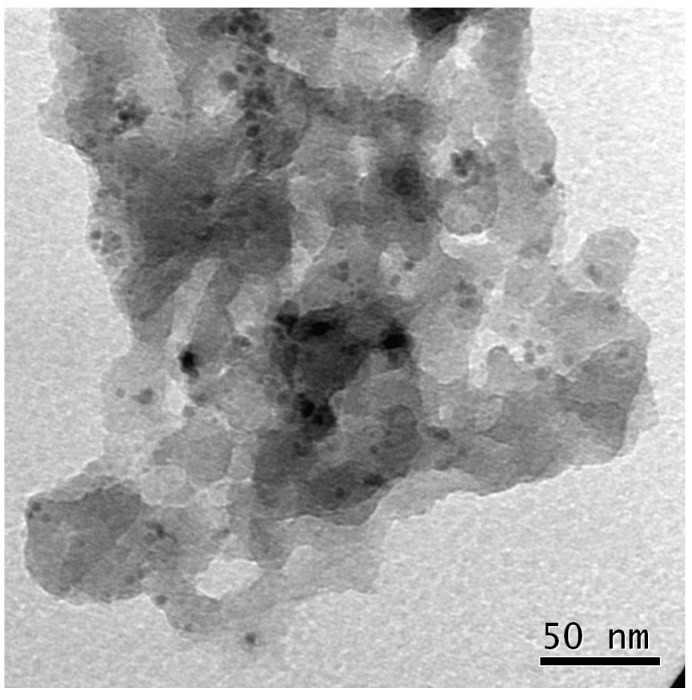
TEM bright field image of the pristine catalyst slice with Pd NPs describing the sponge-like nanostructure of the polymer matrix.

Figure 3 shows a low enlargement TEM image of the pre-catalyst where a sponge-like nanostructure, which is capable of assuring a good contact between the reactive solution and the catalytic centres, is observed. As it will be discussed below, micro-IR and TEM analysis indicated that the structural and chemical features were retained by the polymeric support also after several reaction cycles and no heteroatoms deriving from the reaction substrates and/or products were detected in the porous framework, accordingly to what observed in other preparations [5].

#### 2.3.2. *Pd-Pol* Catalysed Suzuki-Miyaura Cross-Coupling

**Figure 4:**
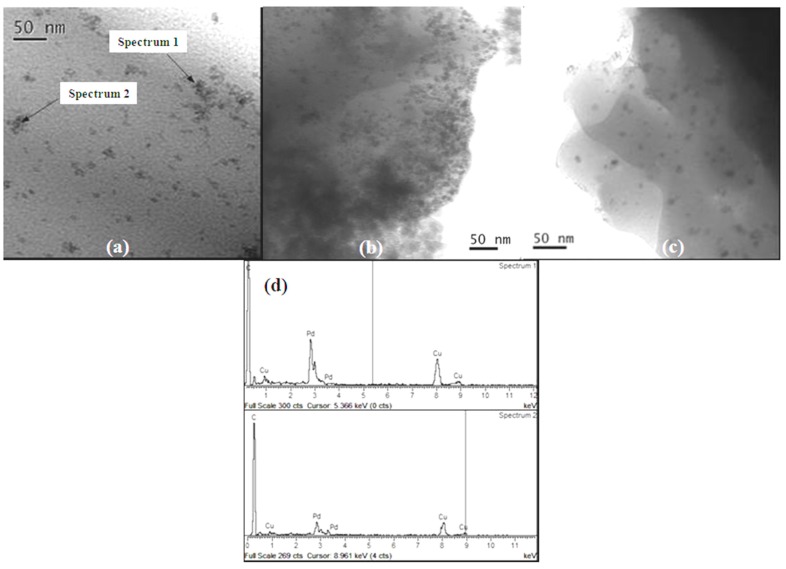
TEM bright field images of *Pd-pol* after (**a**) 30 min; (**b**) the first cycle; and (**c**) the fifth cycle of the Suzuki-Miyaura cross-coupling reaction described in entry 1 of Table 1; (**d**) EDS specta of selected areas of (**a**).

Figure 4 shows TEM images of *Pd-pol* recovered after (a) 30 min of the first cycle (at 30% substrate conversion); (b) the first cycle (2 h) and (c) the fifth cycle of the Suzuki-Miyaura cross-coupling reaction described in entry 1 of Table 1.

TEM analyses ascertained the reduction of the Pd(II) pre-catalyst and the EDS spectra clearly indicated the presence of Pd NPs (on copper grid) and allowed us to follow their morphology with reaction time. Once more, no contamination from other elements was recorded. Pd reduction was both thermal (being the reaction temperature 100 °C) [30] and chemical (being the excess of phenylboronic acid the reductant).

**Figure 5:**
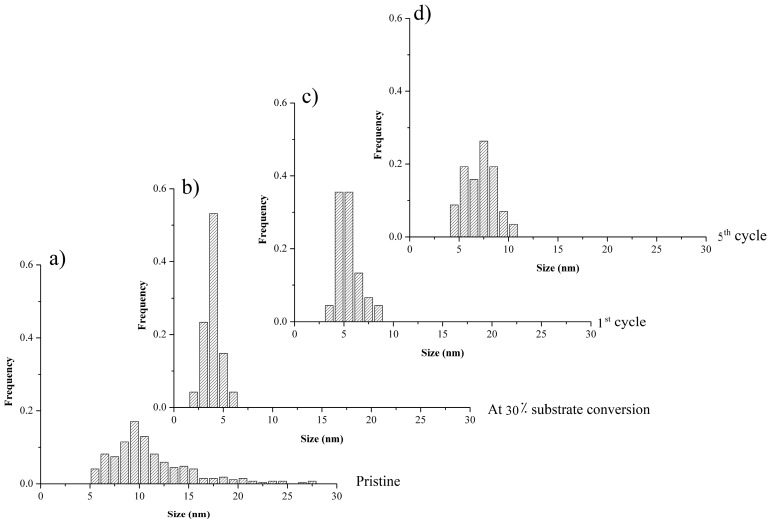
Pd NP size distributions in (**a**) pristine *Pd-pol*and in *Pd-pol*recovered after (**b**) 30 min; (**c**) the first cycle and (**d**) the fifth cycle of the Suzuki-Miyaura cross-coupling reaction described in entry 1 of Table 1.

Figure 5 shows Pd NP size distributions in (a) pristine *Pd-pol* and in *Pd-pol* recovered after (b) 30 min; (c) the first cycle and (d) the fifth cycle of the Suzuki-Miyaura cross-coupling reaction described in entry 1 of Table 1. *Pd-pol* recovered after 30 min reaction (at 30% substrate conversion) contained new Pd NPs whose size ranged from 2 to 6 nm, thus being smaller than the Pd NPs already contained in the pristine catalyst. The same Pd NP size distribution was observed at the end of the reaction (quantitative substrate conversion). Comparing Figure 5c (first cycle) with Figure 5d (fifth cycle) shows that the mean Pd NPs increase with the cycle, with diameters ranging from 2–6 nm to 4–10 nm, and the nanoparticle size distribution becomes broader. Differently from what observed by Mallick *et al.* [70], no particle aggregation was observed, indicating that the reaction temperature was not sufficient to allow atoms diffusion. The nanostructure of the catalyst was substantially maintained during the re-cycles and the Pd NPs remained embedded in the polymer bulk with a higher density of Pd NPs on the support surface, thus pointing to a “release and catch” mechanistic pathway.

The slight decrease in the catalytic activity of *Pd-pol* observed in the Suzuki-Miyaura coupling reaction after the second catalytic cycle (entry 1 of Table 1) can hence be influenced by the increase of the supported nanoparticle sizes during catalysis which may either render the nanoparticles less prone to be leached out in solution, or may result in the leaching of bigger, and therefore less active, nanoparticles.

**Figure 6:**
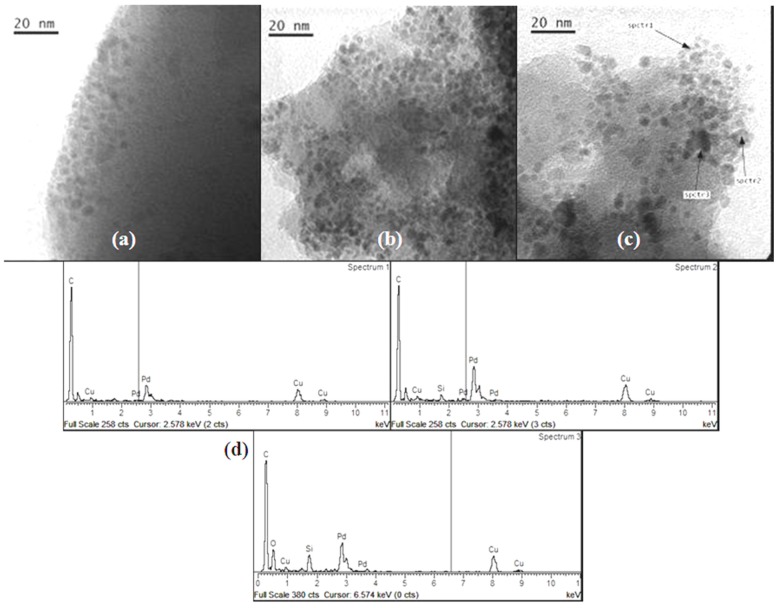
TEM bright field images of *Pd-pol* after (**a**) 5 min of the first cycle; (**b**) the first cycle and (**c**) the fifth cycle of the TBAB assisted Suzuki-Miyaura cross-coupling reaction described in entry 2 of Table 1; (**d**) EDS spectra of selected areas of (**c**) (irregular contamination of silica was observed in Spectrum 3).

Figure 6 shows TEM images indicating the snapshots of the PdNPs formation after (a) 5 min (at 30% substrate conversion); (b) the first cycle and (c) the fifth cycle of the TBAB assisted Suzuki-Miyaura cross-coupling reaction (conditions described in entry 2 of Table 1).

Both *Pd-pol* samples recovered after duty in the Suzuki-Miyaura reaction carried out in the presence and in the absence of TBAB, respectively, presented the same nanostructure. In particular, an increased number of well distributed metal NPs with reduced aggregation during time and the catalytic cycles were observed by TEM analyses. All particles had similar diameters indicating the same growth rate. Some aggregation could be observed for a total number of few primary particles, being the aggregate diameter lesser than 20 nm.

**Figure 7:**
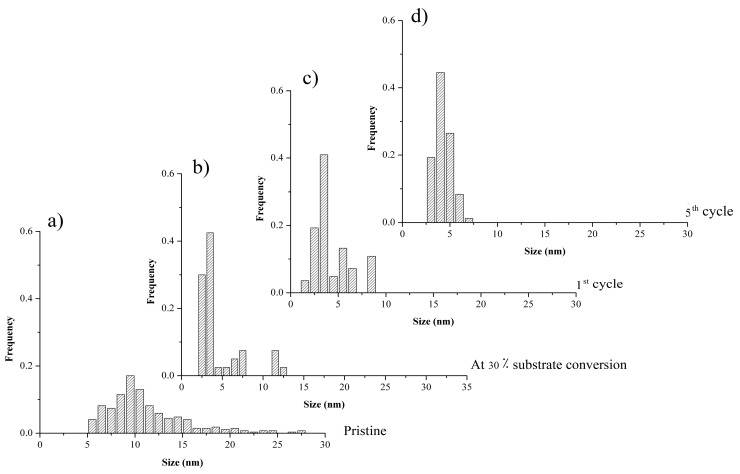
Pd NPs size distributions in (**a**) pristine *Pd-pol* and in *Pd-pol* recovered after (**b**) 5 min; (**c**) the first cycle and (**d**) the fifth cycle of the TBAB assisted Suzuki-Miyaura cross-coupling reaction described in entry 2 of Table 1.

Figure 7 shows Pd NP size distributions in (a) pristine *Pd-pol* and in *Pd-pol* recovered after (b) 5 min; (c) completion of the first cycle and (d) the fifth cycle of the TBAB assisted Suzuki-Miyaura cross-coupling reaction described in entry 2 of Table 1. Pd NP sizes are smaller than the sizes of Pd NPs formed during the cross-coupling reaction carried out in the absence of TBAB.

In particular, after the first TBAB-assisted reaction cycle, a local nucleation of the finer Pd NPs was observed as two dimensional ranges of nanoparticles, the first one with a size distribution centered around 3 nm and the second one with a size distribution centered at 6 nm. The smaller size of Pd NPs obtained in the presence of TBAB as phase transfer agent justified in part the higher catalytic activity of *Pd-pol* with respect to the same reaction carried out in the absence of TBAB (Table 1). However, in the presence of TBAB the “release and catch” process of Pd active species during catalysis occurred mainly for the first part of the phenomenon, being the metal leaching not negligible as revealed by elemental analyses results [45]. This could be due to the strong stabilization capacity of tetraalkylammonium salts towards metal nanoparticles dispersed in solution. On the contrary, in the absence of TBAB, Pd NPs are less prone to be leached out in solution and are more inclined to be re-captured at the end of reaction by the polymer support, which acts as stabilizer. TEM images of the recovered catalyst confirm this hypothesis, showing a higher density of Pd NPs on the surface respect to the inner part of the resin, thus revealing that the Pd NPs re-capturing process by the polymer matrix occurs in the Suzuki reaction carried out in water with (in less extent) and without TBAB.

#### 2.3.3. *Pd-Pol* Catalyst for Nitroarene Reduction with NaBH_4_ and H_2_

**Figure 8:**
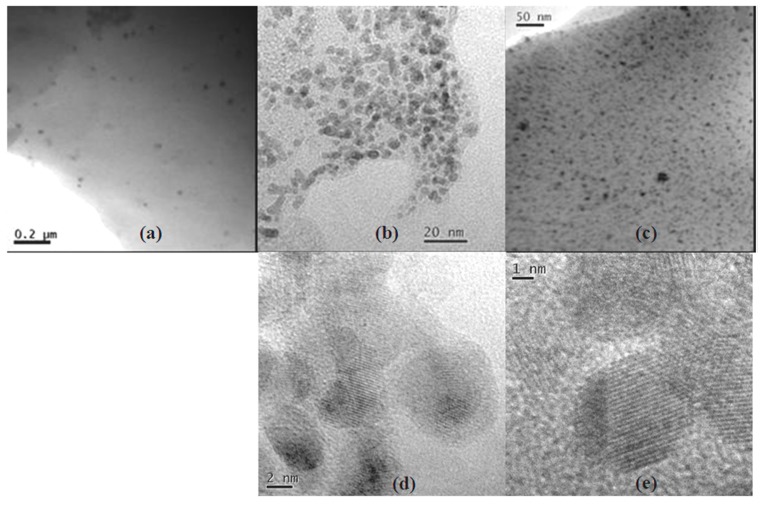
TEM images of *Pd-pol* (**a**) pristine; (**b**) after the first cycle and (**c**) after the twelfth cycle of nitrobenzene reduction with NaBH_4_, described in Table 2; (**d**) HRTEM image of (**b**) and (**e**) HRTEM image of (**c**).

TEM images of *Pd-pol* (a) pristine; (b) after the first cycle and (c) after the twelfth cycle of nitrobenzene reduction with NaBH_4_ are shown in Figure 8.

**Figure 9:**
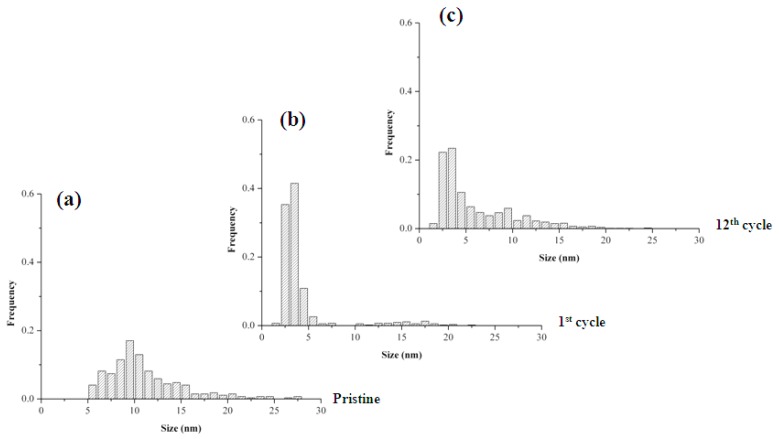
Pd NPs particle size distributionsof *Pd-pol* (**a**) before; (**b**) after the first cycle and (**c**) after the twelfth cycle of nitrobenzene reduction with NaBH_4_.

On passing from the pristine to the first cycle, the recovered *Pd-pol* showed both the previously formed palladium nanoparticles and an increasing high number density of newly formed Pd NPs, now ranging from 2 to 4 nm. As a consequence of such reactivity, Pd NPs showed a bimodal size distribution: a first peak with a mean diameter centred at around 3 nm and the second one centred at *ca.* 10–12 nm (see Figure 9). Remarkably, in *Pd-pol* recovered after the twelfth run, the presence of large (10–12 nm diameter) together with small (diameter equal to *ca.* 3 nm) palladium nanoparticles (Figure 9) did not cause a significant decrease in the catalytic activity, because the smallest Pd NPs were still abundant. High resolution (HR)TEM images showed lattice pattern with an interplanar spacing of 0.22 nm ascribable to metallic Pd {1 1 1} plane [71] as depicted in Figure 8d,e, indicating the single crystallinity nature for these Pd NPs. Attempts to obtain HRTEM images of Pd NP lattice pattern also in *Pd-pol*samples used in the Suzuki-Miyaura reaction were unsuccessful. The absence of the Pd NP crystallinity features of the former *Pd-pol* samples might be ascribable to the presence of a thick layer of amorphous polymeric matrix.

**Figure 10:**
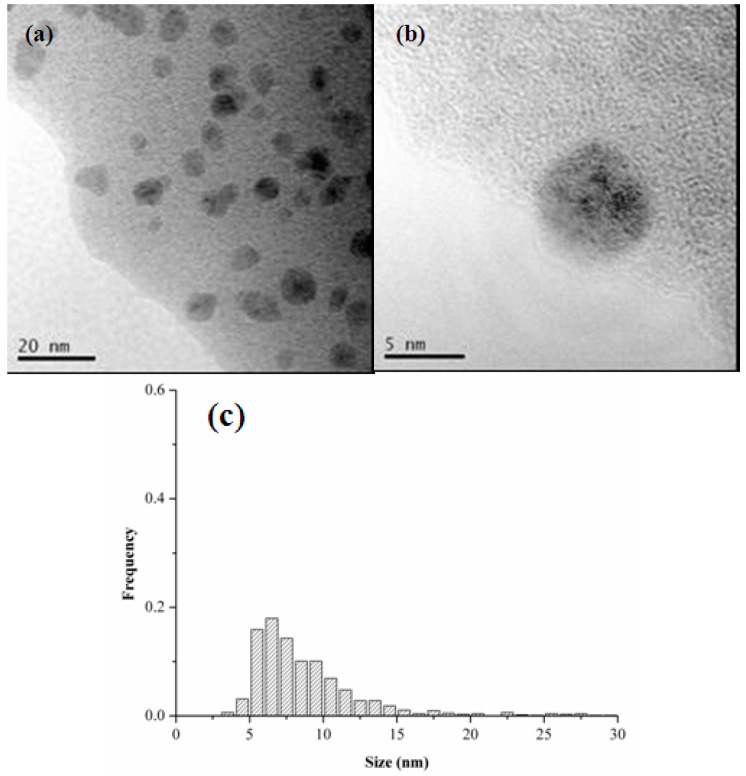
(**a**,**b**) TEM images and (**c**) Pd NPs particle size distribution of *Pd-pol* used in the nitrobenzene reduction under hydrogen at room temperature.

As expected, the formation of catalytically active Pd NPs was observed also when the catalyst was employed for the hydrogenation of nitrobenzene in water under 1 atm H_2_, instead of NaBH_4_ (Figure 10). These nanoparticles, generated by hydrogen reduction, were bigger (6–10 nm diameter sizes) compared to those formed using NaBH_4_ as reducing agent, and consisted of single crystallites with an interplanar spacing of 0.22 nm ascribable again to Pd metallic {1 1 1} plane [71]. It is known that the nature of the reducing agent can strongly influence the nanostructure and the size distribution of the formed metal nanoclusters [71]. The different NPs size distributions observed in the two *Pd-pol* samples, the first one reduced by NaBH_4_ and the second one reduced by 1 atm H_2_, were presumably responsible for the different catalytic activities showed by the two resins (the former system being more active than the latter one). NaBH_4_ acts as a reducing agent of the metal in a dual way, by providing hydrides and by generating hydrogen gas. In fact, the hydrides from sodium borohydride can replace negatively charged ligands bound to Pd(II) (in the present case: the β-ketoesterato moieties, Scheme 1), generating Pd-hydrides which, upon H_2_ elimination, give the reduced metal.

**Scheme 4:**
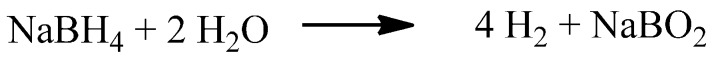
Metal catalyzed production of H_2_ by NaBH_4_ in water.

In addition, the H_2_ formed *in situ* in water (Scheme 4) can reduce Pd(II) to Pd(0) with formation of small size metal nanoparticles. On the contrary, in the case of *Pd-pol* stirred under 1 atm H_2_, the reduction occurs only by hydrogen gas, which has to be transported to the water phase and then by solid phase diffusion to the *Pd-pol* structure. Since the metal reduction by H_2_ requires longer times than the analogous process induced by NaBH_4_, larger palladium crystallites are formed in the reactions carried out under dihydrogen gas.

#### 2.3.4. *Pd-Pol* Catalyst for Quinoline Reduction Using NaBH_4_

**Figure 11:**
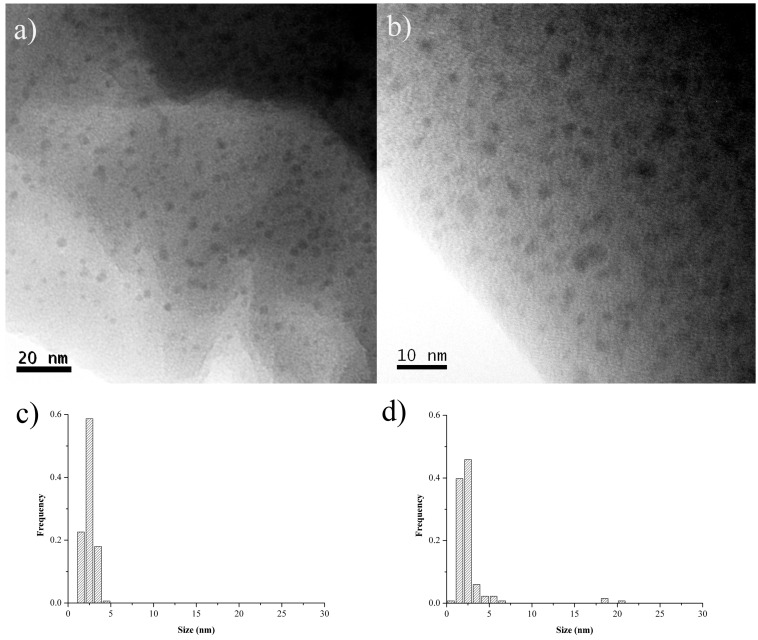
TEM high enlargement images of *Pd-pol* after the (**a**) first and (**b**) seventh cycle of the quinoline reduction using NaBH_4_ at 60 °C in water. Pd NPs particle size distributions of *Pd-pol* after the (**c**) first and (**d**) seventh cycle of the quinoline reduction using NaBH_4_ at 60 °C in water (reaction conditions reported in Table 4).

*Pd-pol* was also tested in the transfer hydrogenation of quinolines to 1,2,3,4-tetrahydroquinolines. Figure 11 shows TEM images of *Pd-pol* recovered after (a) the first and (b) the seventh cycle of the quinoline reduction using NaBH_4_ in water. The number of primary particles in *Pd-pol* recovered after the first cycle resulted higher compared to those observed in the pre-catalyst, and they remained homogeneously distributed throughout the polymer indicating limited diffusion growth mechanism. As occurred in the nitroarene reduction, the Pd NPs had a small average diameter ranging from 2 to 4 nm. The Pd NPs were not grouped in clusters but were individually distributed. On passing from the first (Figure 11a) to the seventh cycle (Figure 11b), the number of smallest size (2 nm) Pd NPs increased, the nanostructure of the catalyst was substantially maintained during the subsequent cycles and the Pd NPs remained embedded into the polymer bulk. The observation of a homogeneous dispersion of the metal NPs onto the polymeric bulk rules out a “release and catch” process during catalysis.

Indeed, TEM results are consistent with a truly heterogeneous mechanism probably assisted by the polymeric support, as depicted in Scheme 3. The clusters of primary nanoparticles did not grow in size, indicating absence of particle mobility under reaction conditions. The Pd NPs supported onto *Pd-pol* remained isolated from each other and they did not aggregate with recycling, although a limited amount of them grew slightly. This explains why the catalytic activity of the supported catalyst recovered after seven cycles was comparable to that of the catalyst reused after the first run.

**Figure 12:**
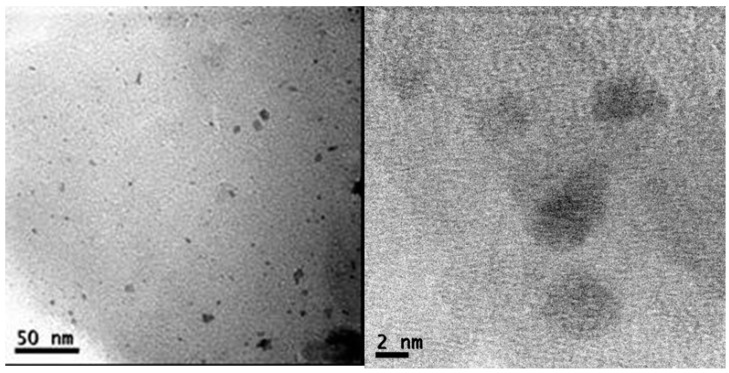
TEM images of *Pd-pol* subjected to the same reaction conditions reported in Table 3 using NaBH_4_ but without substrate.

In order to evaluate the influence of the substrate on the morphological nanostructure of the catalyst, *Pd-pol* was subjected to the same reaction conditions reported in Table 3 using NaBH_4_ in the absence of the substrate. The morphological features of *Pd-pol* recovered after this test were similar to those of *Pd-pol* recovered after the first cycle using the substrate, as shown by the TEM image reported in Figure 12. This indicates a negligible influence of the substrate on the morphological features of the polymer supported Pd NPs.

## 3. Experimental Section

### 3.1. General Considerations

Tap water was deionized by ionic exchange resins (Millipore, Billerica, MA, USA) before use. All other chemicals were purchased from commercial sources and used as received. Palladium content in *Pd-pol* was assessed after sample mineralization by atomic absorption spectrometry using a PerkinElmer 3110 instrument (Perkin-Elmer Corporation, Waltham, MA, USA). Reactions were monitored by GLC or by GC-MS. GC-MS data (EI, 70 eV) were acquired on a HP 6890 instrument (Hewlett Packard, Palo Alto, CA, USA) using a HP-5MS crosslinked 5% PH ME siloxane (30.0 m × 0.25 mm × 0.25 mm) capillary column coupled with a mass spectrometer HP 5973. GLC analysis of the products was performed using a HP 6890 instrument equipped with a FID detector and a HP-1 (crosslinked methylsiloxane) capillary column (60.0 m × 0.25 mm × 1.0 μm). Pd(AAEMA)_2_ and the supported catalyst (*Pd-pol*) were prepared as reported in [55]. The procedure adopted for the Suzuki-Miyaura reaction catalyzed by *Pd-pol* is reported in [45]. The reductions of nitrobenzene were carried out as reported in [46]. The quinoline reduction using NaBH_4_ was carried out as reported in [4].

### 3.2. Morphological Analyses

*Pd-pol* was recovered after duty by filtration (in the case of Suzuki-Miyaura reaction) or by centrifugation (in all other cases). The isolated catalyst was then repeatedly washed with water, acetone, and diethyl ether and dryed under high vacuum.

The microstructure of the polymeric matrix embedded NPs was determined by TEM observations at acceleration voltage of 200 kV (Model JEM 2010, Jeol, Akishima, Tokyo, Japan), equipped with X-ray energy dispersive spectroscopy (EDS, Plano, TX, USA). The samples were prepared by dispersing the powders in distilled water using an ultrasonic stirrer and then placing a drop of suspension on a copper grid covered with a transparent polymer film, followed by drying and carbon coating. The particle size distributions were obtained by TEM image analysis using the ImageJ software [72]. Each morphology observation was accompanied by EDS analyses that confirmed the presence of Pd. Micro-FTIR spectra (neat) were recorded using a PerkinElmer Spectrum 2000 instrument.

## 4. Conclusions

A morphological study on Pd NPs supported onto a polymer matrix (*Pd-pol*) was carried out by TEM, EDS and micro-IR techniques. These analyses were performed on such material before, during and after its exposure to prolonged catalytic runs under green conditions in water solvent in the following organic reactions: the Suzuki-Miyaura coupling between aryl bromides and phenylboronic acid and the hydrogenation of nitroarenes and quinolines, using dihydrogen or NaBH_4_, as reducing agent. This study shed light into morphological features of both metal NPs and polymeric insoluble support. Micro-IR analyses revealed that the support was chemically stable over the recycles of the catalyst in all the reactions tested, while TEM techniques showed that the macroporosity of the resin remained constant after each run (with good swelling in water), an important requirement for insoluble catalytic materials capable of facilitating the migration of reactants towards active sites.

Moreover, TEM analyses showed that in all cases the pristine Pd(II) polymer supported complex (the pre-catalyst) was reduced *in situ* to Pd(0) forming nanoparticles (the real active species) under the reaction conditions. The organic polymer was always able to stabilize Pd NPs during recycling, since metal NPs agglomeration detected by TEM analysis was always negligible. In all cases, after several re-uses (ranging from 5 to 12, depending on the reaction) the amount (as a percent) of the smallest Pd NPs (2–8 nm diameter) still remained high, while the presence of larger NPs (10–15 nm diameter) might indicate growth (no agglomeration) of the smallest Pd NPs with recycling. However, the level of NP growth that we did observe with reuse was not enough to cause a significant decrease of the catalytic activity.

The Pd NPs’ average size and morphology was affected by several parameters, such as: temperature, presence of a tetralkylammonium salt, and reducing agent. High temperatures (80–100 °C) favoured the thermal reduction of Pd(II) with formation of NPs of 9 nm average size, while at room temperature a greater amount of smaller NPs (2–6 nm diameter) was observed.

The presence of a phase transfer agent (tetrabutylammonium bromide) caused the formation of smaller size Pd NPs (4 nm average size) and metal leaching into solution, due to its well-known metal NPs stabilizing capacity.

TEM observations allowed us to deduce the following considerations about the effect of the reducing agent on size and morphology of Pd NPs. In the case of the Suzuki coupling, the effect of the reductant (excess phenylboronic acid) was masked by the temperature reaction (100 °C) and/or by the presence of tetrabutylammonium bromide (when used). On the contrary, for the reduction of nitroarenes and quinolines in water the choice of the reductant (H_2_ or NaBH_4_) was crucial for Pd NPs diameter size (and activity), ranging from 6 to 10 nm under 1 atm dihydrogen and from 2 to 4 nm, in the presence of NaBH_4_, because the latter caused the reduction of Pd(II) in a more effective way.

Finally, morphological features of the supported catalyst could provide important information about the mechanism involved in the catalytic cycle. The “release and catch” process of active species, commonly invoked by the scientific community for the heterogeneously catalysed C–C bond forming reaction could explain why TEM images of *Pd-pol* recovered after duty in the Suzuki reaction showed the majority of Pd NPs onto the surface of the polymer. On the contrary, when *Pd-pol* was employed in the hydrogenation under H_2_ and in the NaBH_4_ transfer hydrogenation of nitroarenes and quinolines, TEM images showed a uniform distribution of Pd NPs in the polymer matrix, pointing to a truly heterogeneous catalytic mechanism, as confirmed by other techniques (hot filtration test, ICP analyses, *etc.*).

All these results offer interesting perspectives in catalysis, since it is well documented that the catalytic activity of metal NPs depends on morphological features, on size control and size distribution. Finally, the present work demonstrates that, when dealing with the characterization of polymer supported metal NPs, a suitable combination of multiple complementary techniques may allow one to obtain a clear picture, not only of the entire process that leads from the metal ion precursor to metal NPs, but also of the catalytic cycle promoted by the metal NPs catalyst.

## References

[1] Astruc D., Lu F., Aranzaes J.R. (2005). Nanoparticles as recyclable catalysts: The frontier between homogeneous and heterogeneous catalysis. Angew. Chem. Int. Ed..

[2] Yan N., Xiao C., Kou Y. (2010). Transition metal nanoparticle catalysis in green solvents. Coord. Chem. Rev..

[3] Fihri A., Bouhrara M., Nekoueishahraki B., Basset J.-M., Polshettiwar V. (2011). Nanocatalysts for Suzuki cross-coupling reactions. Chem. Soc. Rev..

[4] Sarkar S., Guibal E., Quignard F., SenGupta A.K. (2012). Polymer-supported metals and metal oxide nanoparticles: Synthesis, characterization, and applications. J. Nanopart. Res..

[5] Ishida T., Onuma Y., Kinjo K., Hamasaki A., Ohashi H., Honma T., Akita T., Yokoyama T., Tokunaga M., Haruta M. (2014). Preparation of microporous polymer-encapsulated Pd nanoparticles and their catalytic performance for hydrogenation and oxidation. Tetrahedron.

[6] Cooper A.I. (2009). Conjugated microporous polymers. Adv. Mater..

[7] Ding S.-Y., Wang W. (2012). Covalent organic frameworks (COFs): From design to applications. Chem. Soc. Rev..

[8] Feng X., Ding X., Jiang D. (2012). Covalent organic frameworks. Chem. Soc. Rev..

[9] Kalidindi S.B., Oh H., Hirscher M., Esken D., Wiktor C., Turner S., van Tendeloo G., Fischer R.A. (2012). Metal@COFs: Covalent organic frameworks as templates for Pd nanoparticles and hydrogen storage properties of Pd@COF-102 hybrid material. Chem. Eur. J..

[10] Hermes S., Schröter M.-K., Schmid R., Khodeir L., Muhler M., Tissler A., Fischer R.W., Fischer R.A. (2005). Metal@MOF: Loading of highly porous coordination polymers host lattices by metal organic chemical vapor deposition. Angew. Chem. Int. Ed..

[11] Hermes S., Schröder F., Amirjalayer S., Schmid R., Fischer R.A. (2006). Loading of porous metal–organic open frameworks with organometallic CVD precursors: Inclusion compounds of the type [L_n_M]_a_@MOF-5. J. Mater. Chem..

[12] Schröder F., Esken D., Cokoja M., van den Berg M.W.E., Lebedev O.I., van Tendeloo G., Walaszek B., Buntkowsky G., Limbach H.-H., Chaudret B. (2008). Ruthenium nanoparticles inside porous [Zn_4_O(bdc)_3_] by hydrogenolysis of adsorbed [Ru(cod)(cot)]: A solid-state reference system for surfactant-stabilized ruthenium colloids. J. Am. Chem. Soc..

[13] Ishida T., Nagaoka M., Akita T., Haruta M. (2008). Deposition of gold clusters on porous coordination polymers by solid grinding and their catalytic activity in aerobic oxidation of alcohols. Chem. Eur. J..

[14] Ishida T., Kinoshita N., Okatsu H., Akita T., Takei T., Haruta M. (2008). Influence of the support and the size of gold clusters on catalytic activity for glucose oxidation. Angew. Chem. Int. Ed..

[15] Ishida T., Kawakita N., Akita T., Haruta M. (2009). One-pot N-alkylation of primary amines to secondary amines by gold clusters supported on porous coordination polymers. Gold Bull..

[16] Ishida T., Watanabe H., Bebeko T., Akita T., Haruta M. (2010). Aerobic oxidation of glucose over gold nanoparticles deposited on cellulose. Appl. Catal. Gen..

[17] Ishida T., Takamura R., Takei T., Akita T., Haruta M. (2012). Support effects of metal oxides on gold-catalyzed one-pot *N*-alkylation of amine with alcohol. Appl. Catal. Gen..

[18] Hasell T., Wood C.D., Clowes R., Jones J.T.A., Khimyak Y.Z., Adams D.J., Cooper A.I. (2010). Palladium Nanoparticle incorporation in conjugated microporous polymers by supercritical fluid processing. Chem. Mater..

[19] Schmidt J., Weber J., Epping J.D., Antonietti M., Thomas A. (2009). Microporous conjugated poly(thienylene arylene) networks. Adv. Mater..

[20] Chan-Thaw C.E., Villa A., Prati L., Thomas A. (2011). Triazine-based polymers as nanostructured supports for the liquid-phase oxidation of alcohols. Chem. Eur. J..

[21] Chan-Thaw C.E., Villa A., Katekomol P., Su D., Thomas A., Prati L. (2010). Covalent Triazine framework as catalytic support for liquid phase reaction. Nano Lett..

[22] Chan-Thaw C.E., Villa A., Veith G.M., Kailasam K., Adamczyk L.A., Unocic R.R., Prati L., Thomas A. (2012). Influence of periodic nitrogen functionality on the selective oxidation of alcohols. Chem. Asian J..

[23] Palkovits R., Antonietti M., Kuhn P., Thomas A., Schüth F. (2009). Solid Catalysts for the selective low-temperature oxidation of methane to methanol. Angew. Chem. Int. Ed..

[24] Ogasawara S., Kato S. (2010). Palladium nanoparticles captured in microporous polymers: A tailor-made catalyst for heterogeneous carbon cross-coupling reactions. J. Am. Chem. Soc..

[25] Yang Y., Ogasawara S., Li G., Kato S. (2013). Water compatible Pd nanoparticle catalysts supported on microporous polymers: Their controllable microstructure and extremely low Pd-leaching behaviour. J. Mater. Chem. A.

[26] Anna M., Romanazzi G., Mastrorilli P. (2013). Polymer supported catalysts obtained from metal-containing monomers. Curr. Org. Chem..

[27] Dzhardimalieva G.I., Dorokhov V.G., Golubeva N.D., Pomogailo S.I., Lyakhovich A.M., Savchenko V.I., Pomogailo A.D. (2010). Reactivity of metal-containing monomers 66. Hydrogenation of nitrotoluene derivatives in the presence of polymer-immobilized Pd nanoparticles. Russ. Chem. Bull..

[28] Dell’Anna M.M., Mastrorilli P., Rizzuti A., Suranna G.P., Nobile C.F. (2000). Synthesis and copolymerization of rhodium(I) and palladium(II) complexes with the deprotonated form of 2-(acetoacetoxy)ethyl methacrylate. Inorg. Chim. Acta.

[29] Dell’Anna M.M., Mastrorilli P., Nobile C.F., Scott P.J.H. (2012). Solid-Phase Catalytic Activity of a polymer-supported palladium complex. Solid-Phase Organic Syntheses.

[30] Rozenberg A.S., Rozenberg A.A., Dzardimalieva G.I., Pomogailo A.D. (2005). Formation of metal-containing nanoparticles in polymer matrix. Computer simulation of clusterization kinetics during the solid-phase thermal decomposition of metal-containing precursors. Colloid J..

[31] Dell’Anna M.M., Gallo V., Mastrorilli P., Romanazzi G. (2010). A Recyclable nanoparticle-supported rhodium catalyst for hydrogenation reactions. Molecules.

[32] Pomogailo S.I., Dzhardimalieva G.I., Pomogailo A.D. (2008). Polymerization and catalytic properties of cluster-containing monomers and polymers. Macromol. Symp..

[33] Li Y., Hong X.M., Collard D.M., El-Sayed M.A. (2000). Suzuki cross-coupling reactions catalyzed by palladium nanoparticles in aqueous solution. Org. Lett..

[34] Narayanan R., El-Sayed M.A. (2003). Effect of catalysis on the stability of metallic nanoparticles:  Suzuki reaction catalyzed by PVP-palladium nanoparticles. J. Am. Chem. Soc..

[35] Durap F., Metin Ö., Aydemir M., Özkar S. (2009). New route to synthesis of PVP-stabilized palladium(0) nanoclusters and their enhanced catalytic activity in Heck and Suzuki cross-coupling reactions. Appl. Organomet. Chem..

[36] Li Y., El-Sayed M.A. (2001). The effect of stabilizers on the catalytic activity and stability of Pd colloidal nanoparticles in the Suzuki reactions in aqueous solution. J. Phys. Chem. B.

[37] Liu Y., Khemtong C., Hu J. (2004). Synthesis and catalytic activity of a poly(*N*,*N*-dialkylcarbodiimide)/palladium nanoparticle composite: A case in the Suzuki coupling reaction using microwave and conventional heating. Chem. Commun..

[38] Molnár Á. (2011). Efficient, selective, and recyclable palladium catalysts in carbon–carbon coupling reactions. Chem. Rev..

[39] Albéniz A.C., Carrera N. (2011). Polymers for green C–C couplings. Eur. J. Inorg. Chem..

[40] Bianchini C., dal Santo V., Meli A., Moneti S., Moreno M., Oberhauser W., Psaro R., Sordelli L., Vizza F. (2003). A comparison between silica-immobilized ruthenium(II) single sites and silica-supported ruthenium nanoparticles in the catalytic hydrogenation of model hetero- and polyaromatics contained in raw oil materials. J. Catal..

[41] Sun B., Khan F.-A., Vallat A., Süss-Fink G. (2013). NanoRu@hectorite: A heterogeneous catalyst with switchable selectivity for the hydrogenation of quinoline. Appl. Catal. Gen..

[42] Barbaro P., Gonsalvi L., Guerriero A., Liguori F. (2012). Facile heterogeneous catalytic hydrogenations of CN and CO bonds in neat water: Anchoring of water-soluble metal complexes onto ion-exchange resins. Green Chem..

[43] Mao H., Chen C., Liao X., Shi B. (2011). Catalytic hydrogenation of quinoline over recyclable palladium nanoparticles supported on tannin grafted collagen fibers. J. Mol. Catal. Chem..

[44] Mao H., Ma J., Liao Y., Zhao S., Liao X. (2013). Using plant tannin as natural amphiphilic stabilizer to construct an aqueous–organic biphasic system for highly active and selective hydrogenation of quinoline. Catal. Sci. Technol..

[45] Dell’Anna M.M., Mali M., Mastrorilli P., Rizzuti A., Ponzoni C., Leonelli C. (2013). Suzuki-Miyaura coupling under air in water promoted by polymer supported palladium nanoparticles. J. Mol. Catal. Chem..

[46] Dell’Anna M.M., Intini S., Romanazzi G., Rizzuti A., Leonelli C., Piccinni F., Mastrorilli P. (2014). Polymer supported palladium nanocrystals as efficient and recyclable catalyst for the reduction of nitroarenes to anilines under mild conditions in water. J. Mol. Catal. Chem..

[47] Dell’Anna M.M., Capodiferro V.F., Mali M., Manno D., Cotugno P., Monopoli A., Mastrorilli P. (2014). Highly selective hydrogenation of quinolines promoted by recyclable polymer supported palladium nanoparticles under mild conditions in aqueous medium. Appl. Catal. Gen..

[48] Dell’Anna M.M., Romanazzi G., Intini S., Rizzuti A., Leonelli C., Piccinni A.F., Mastrorilli P. (2015). A polymer supported palladium(II) β-ketoesterate complex as active and recyclable pre-catalyst for selective reduction of quinolines in water with sodium borohydride. J. Mol. Catal. Chem..

[49] Benaglia M. (2006). Recoverable and recyclable chiral organic catalysts. New J. Chem..

[50] Dell’Anna M.M., Gagliardi M., Mastrorilli P., Suranna G.P., Nobile C.F. (2000). Hydrogenation reactions catalysed by a supported palladium complex. J. Mol. Catal. Chem..

[51] Dell’Anna M.M., Mastrorilli P., Muscio F., Nobile C.F., Suranna G.P. (2002). A polymer-supported β-ketoesterate complex of palladium as an efficient, phosphane-free, air-stable, recyclable catalyst for the Heck reaction. Eur. J. Inorg. Chem..

[52] Dell’Anna M.M., Mastrorilli P., Muscio F., Nobile C.F. (2003). A new polymer supported palladium complex as active, air stable and recyclable catalyst for carbon-carbon bond forming reactions. Stud. Surf. Sci. Catal..

[53] Dell’Anna M.M., Mastrorilli P., Nobile C.F., Suranna G.P. (2003). Asymmetric allylic alkylation using a polymer-supported palladium catalyst in the presence of chiral ligands. J. Mol. Catal. Chem..

[54] Dell’Anna M.M., Lofù A., Mastrorilli P., Mucciante V., Nobile C.F. (2006). Stille coupling reactions catalysed by a polymer supported palladium complex. J. Organomet. Chem..

[55] Dell’Anna M.M., Mastrorilli P., Rizzuti A., Leonelli C. (2011). One-pot synthesis of aniline derivatives from nitroarenes under mild conditions promoted by a recyclable polymer-supported palladium catalyst. Appl. Catal. Gen..

[56] Dell’Anna M.M., Mali M., Mastrorilli P., Cotugno P., Monopoli A. (2014). Oxidation of benzyl alcohols to aldehydes and ketones under air in water using a polymer supported palladium catalyst. J. Mol. Catal. Chem..

[57] Xia Y., Xiong Y., Lim B., Skrabalak S.E. (2009). Shape-controlled synthesis of metal nanocrystals: Simple chemistry meets complex physics?. Angew. Chem. Int. Ed..

[58] Groppo E., Agostini G., Borfecchia E., Wei L., Giannici F., Portale G., Longo A., Lamberti C. (2014). Formation and growth of Pd nanoparticles inside a highly cross-linked polystyrene support: Role of the reducing agent. J. Phys. Chem. C.

[59] Reetz M.T., Westermann E. (2000). Phosphane-free palladium-catalyzed coupling reactions: The decisive Role of Pd nanoparticles. Angew. Chem. Int. Ed..

[60] Gruttadauria M., Giacalone F., Noto R. (2013). “Release and catch” catalytic systems. Green Chem..

[61] Ohtaka A., Okagaki T., Hamasaka G., Uozumi Y., Shinagawa T., Shimomura O., Nomura R. (2015). Application of “boomerang” linear polystyrene-stabilized Pd nanoparticles to a series of C–C coupling reactions in water. Catalysts.

[62] Bullock R.M. (2004). Catalytic ionic hydrogenations. Chem. Eur. J..

[63] Ren D., He L., Yu L., Ding R.-S., Liu Y.-M., Cao Y., He H.-Y., Fan K.-N. (2012). An unusual chemoselective hydrogenation of quinoline compounds using supported gold catalysts. J. Am. Chem. Soc..

[64] Fang M., Sánchez-Delgado R.A. (2014). Ruthenium nanoparticles supported on magnesium oxide: A versatile and recyclable dual-site catalyst for hydrogenation of mono- and poly-cyclic arenes, *N*-heteroaromatics, and *S*-heteroaromatics. J. Catal..

[65] Fang M., Machalaba N., Sánchez-Delgado R.A. (2011). Hydrogenation of arenes and *N*-heteroaromatic compounds over ruthenium nanoparticles on poly(4-vinylpyridine): A versatile catalyst operating by a substrate-dependent dual site mechanism. Dalton Trans..

[66] De Zan L., Gasparovicova D., Kralik M., Centomo P., Carraro M., Campestrini S., Jerabek K., Corain B. (2007). Nanoclustered palladium(0) supported on a gel-type poly-acrylonitrile–*N*,*N*-dimethylacrylamide–ethylenedimethacrylate resin: Nanostructural aspects and catalytic behaviour. J. Mol. Catal. Chem..

[67] Corain B., Jerabek K., Centomo P., Canton P. (2004). Generation of size-controlled Pd^0^ nanoclusters inside nanoporous domains of gel-type resins: Diverse and convergent evidence that supports a strategy of template-controlled synthesis. Angew. Chem. Int. Ed..

[68] Scalzullo S., Mondal K., Witcomb M., Deshmukh A., Scurrell M., Mallick K. (2008). Polymer-encapsulated metal nanoparticles: Optical, structural, micro-analytical and hydrogenation studies of a composite material. Nanotechnology.

[69] Gasc F., Clerc S., Gayon E., Campagne J.-M., Lacroix-Desmazes P. (2015). Supercritical CO_2_-mediated design of Pd supported catalysts using an amphiphilic functional copolymer. J. Supercrit. Fluids.

[70] Mallick K., Mondal K., Witcomb M., Deshmukh A., Scurrell M. (2008). Catalytic activity of a soft composite material: Nanoparticle location–activity relationship. Mater. Sci. Eng. B.

[71] Yarulin A., Yuranov I., Cárdenas-Lizana F., Abdulkin P., Kiwi-Minsker L. (2013). Size-effect of Pd-(poly(*N*-vinyl-2-pyrrolidone)) nanocatalysts on selective hydrogenation of alkynols with different alkyl chains. J. Phys. Chem. C.

[72] Wayne Rasband. http://rsb.info.nih.gov/ij/.

